# Oral health status of 12-year-old school children in Khartoum state, the Sudan; a school-based survey

**DOI:** 10.1186/1472-6831-9-15

**Published:** 2009-06-15

**Authors:** Nazik Mostafa Nurelhuda, Tordis Agnete Trovik, Raouf Wahab Ali, Mutaz Faisal Ahmed

**Affiliations:** 1 Centre for International Health and Department of Clinical Dentistry, Faculty of Medicine and Dentistry, University of Bergen, Bergen, Norway; 2 Department of Clinical Dentistry, Preventive Dental Care, Faculty of Medicine and Dentistry, University of Bergen, Bergen, Norway; 3 Department of Periodontology, Faculty of Dentistry, University of Science and Technology, Omdurman, Sudan; 4 Restorative Department, Liverpool University Dental Hospital, Liverpool, UK

## Abstract

**Background:**

Few studies have investigated the prevalence of dental caries among school children in the past decades in Sudan rendering it difficult to understand the status and pattern of oral health.

**Methods:**

A school-based survey was conducted using stratified random cluster sampling in Khartoum state, Sudan. Data was collected through interviews and clinical examination by a single examiner. DMFT was measured according to WHO criteria. Gingival index (GI) of Loe & Silness and Plaque index (PI) of Silness & Loe were used.

**Results:**

The mean DMFT for 12-year-olds was found to be 0.42 with a significant caries index (SiC) of 1.4. Private school attendees had significantly higher DMFT (0.57) when compared to public school attendees (0.4). The untreated caries prevalence was 30.5%. In multivariate analysis caries experience (DMFT > 0) was found to be significantly and directly associated with socioeconomic status. The mean GI for the six index teeth was found to be 1.05 (CI 1.03 – 1.07) and the mean PI was 1.30 (CI 1.22 – 1.38).

**Conclusion:**

The prevalence of caries was found to be low. The school children with the higher socioeconomic status formed the high risk group.

## Background

The Sudan, largest African country by size, has more than 500 ethnic groups with diversity in language and culture. Khartoum state alone has an estimated population of 6.2 million describing 16.7% of the approximated 37.2 million total population with 37.6% living in urban areas. Children less than 15-years of age are estimated to be 41.3% of the total inhabitants [1].

Worldwide, dental caries is the most prevalent of the oral diseases with considerable variations in its occurrence between countries, regions within countries, areas within regions and within social and ethnic groups [2]. A few studies investigated the prevalence of dental caries in the Sudan in the past two decades. In 1986, DMFT values were found to be 2.9, 3.2 and 2.3 in 12-year-old urban and rural and 11-year-old semi urban children, respectively [3]. Two years later, another study found that the DMFT rose to 3.2 in the total sample examined [4]. In 1993, Raadal et al. found the mean dmft to be 1.68 in the preschool group and 2.77 in the school group. However, the mean DMFS was 2.08 in the preschool and 3.78 in the school group [5]. All the above investigations were conducted in Khartoum city. Through this study we intend to update the accumulating data worldwide in keeping with the international trend on the index age group of 12-year-olds.

### Education in the Sudan

Education in public schools is officially funded by the government. Schools are concentrated in urban areas where Arabic is the medium of instruction in all public schools and most private schools. The estimated primary school enrolment in Sudan was 54% of the eligible pupils in 2007 [6]. Enrolment varies widely with as high as 78% in Khartoum city. The majority of the primary school children in Khartoum city attend public schools (88%) while the remaining (12%) attend private schools as of the census of 2006 [7]. This distribution justified the assumption that most 12-year-old children in Khartoum state could be found in schools.

The aims of this paper were to assess the general oral health status of the 12-year-old school children in Khartoum state and to determine risk indicators associated with poor oral health status.

## Methods

### Sampling method

**Figure 1:**
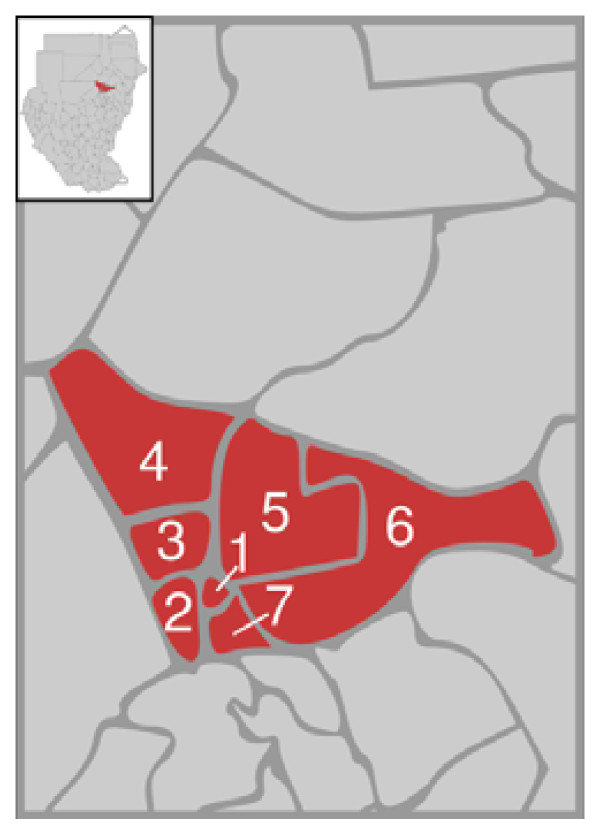
**Localities of Khartoum state, Republic of the Sudan**. 1: Khartoum 2: Ombada, 3: Omdurman, 4: Karary, 5: Bahri, 6: Sharq Elnil, 7: Jabal Awliya.

A school-based survey was conducted in Khartoum state which is divided into 7 main localities (*Khartoum, Jabal Awliya, Omdurman, Ombada, Karary, Bahry and Sharq Elnil*) Figure 1. A two-stage probability proportional to size cluster sampling technique was used [8], taking into consideration school sector (private and public), the school density and the distribution of girls and boys in each locality. The sample size was calculated by applying an estimated dental caries prevalence of 50%, a design effect of 2, and a precision of 0.06. The minimum sample size to satisfy these requirements was estimated to be 550 children in each school sector with dropouts taken into account.

Thus, 37 schools were selected at random from lists obtained from the Ministry of Education in Khartoum. These were 8 public boys' schools, 8 public girls' schools, 5 public mixed gender schools, 8 private boys' schools and 8 private girls' schools. The children were randomly selected from each class. The desired number of children was not always found complete in the randomly selected schools. On that account, more schools were chosen with the geographical proximity being the criteria of selection. A total of 58 schools were eventually visited. Only one of the randomly selected schools was located in a site previously reported to have a water source with high fluoride content.

The sample included healthy (present in school and free from any serious illness) 12-year-old school children who had not experienced multiple extractions (more than 5 missing teeth). The child's age was confirmed from the school registries; otherwise self-reported. School children were selected randomly from any of the classes, ranging from grade 2 in remote public schools to grade 8 in private schools in the city centre.

The final sample comprised 1,109 school children; 49.9% boys (n = 553) and 50.1% girls (n = 556). This sample comprised two statistically comparable groups (public/private proportion was approximately 1:1) of school children attending public (n = 557) and private schools (n = 552). The simple joint result of these groups is referred to as the "total" sample in the forthcoming results. Furthermore, to report results that may represent the 12-year-old school children population in Khartoum state, the afore mentioned groups were weighted according to their distribution in the area (public/private proportion was approximately 7:1). These results are reported under the "representative" group.

### Data collection

This survey was conducted from October 2007 to February 2008. Calibration exercises were carried out at the University of Bergen. The questionnaires were constructed in English translated to Arabic and back translated for validation. A pilot study was carried out. Consequently, implemented changes included a shift from self-administered to a face-to-face interview of the children in a classroom setting. When the child was unable to report on parents' education level, a letter was given to the child to be completed by their parents and returned to the author (NMN) to this effect.

All the children were clinically examined by the main researcher (NMN) accompanied by a trained assistant who recorded the data on a standardized form. The examination was carried out under natural sunlight using a disposable plane mirror and dental explorer. The later was used to assess the plaque accumulation on the buccal tooth surface. The child was made to lie down on his back on a table or a bench and the examiner at its head. Only completely erupted permanent teeth were included. The children were given oral hygiene instructions following the examination and rewarded with a toothpaste tube. Children who needed treatment were referred to the closest dental care facility.

### Measurements

#### Clinical measures

The clinical oral health status was measured using the decayed, missing and filled tooth index (DMFT) according to the WHO caries diagnostic criteria for epidemiological studies [9]. A tooth was marked as 'decayed' when any of the following was observed: unmistakable cavitations on the occlusal, buccal or lingual walls of the tooth, a detectable softened floor or wall or remaining, carious roots and filled tooth with signs of caries. When in doubt, the tooth was recorded as sound. A tooth extracted due to caries was marked 'missing'. *Significant caries index *SiC was calculated as the mean DMFT of the one third of the study group with the highest caries score [10]. The *care index *was defined as the fraction of filled teeth (FT) to DMFT [11].

The gingival index (GI) of Loe and Silness (1963) [12] and the plaque index (PI) of Silness and Loe (1964) [13] were used. GI and PI were scored based on a 0–3 scale that combines an assessment of tissue colour and form with bleeding on stimulation assessment and presence of plaque, where 0 represented healthy gingiva and plaque free tooth surface respectively. Buccal surfaces of all the present teeth were examined. When in doubt between scoring 0 or 1, the score 1 was given. Dean's index was used to record the prevalence of dental fluorosis [14]. Results are reported as either sound (score 0) or fluorosed (scores 1–4: mild to severe).

#### Sociodemographic characteristics

The survey comprised questions on dichotomous indicators of socioeconomic status (SES) such as parental education. Father's and mother's education were organised into 'lower group' – no formal education, 'middle group' – parents who attended primary and secondary schools and 'higher group' – university and beyond education. Father's education was furthermore dichotomized for Poisson regression analysis into lower (lower and middle groups) and higher (higher group). Localities were grouped into two, according to their proximity to the capital city; urban -Khartoum, Bahri and Omdurman and rural- Jabal Awliya, Sharq Elnil, Ombada and Karary Table 1.

**Table 1:** Frequency distribution (%, n) according to sociodemographic characteristics and dental treatment availability among school children in Khartoum state, the Sudan

**Sociodemographic characteristics**	**Public schools** **%(n)**	**Private schools %(n)**	**Total sample %(n)**	***Representative*** **sample %** *	**P** **Value #**
**Father's education**					
Low	19.9 (111)	4.2 (23)	12.0 (134)	18.1	
Medium	52.2 (291)	28.6 (158)	40.3 (449)	49.4	
High	26.9 (150)	66.7 (368)	46.5 (518)	31.7	.000
**Mother's education**					
Low	23.3 (130)	3.6 (20)	13.5 (150)	21.0	
Medium	62.5 (348)	54.7 (302)	58.3 (650)	61.6	
High	13.6 (76)	40.6 (224)	26.9 (300)	16.9	.000
**Locality**					
Urban	28.4 (158)	64.9 (358)	46.5 (516)	32.8	.000
**Socioeconomic status variable**					
Middle	21.2 (118)	50.2 (277)	35.4 (395)	24.6	.000
**History of dentist visit**					
Follow-up & checkup	1.1 (6)	3.3 (18)	2.2 (24)	1.3	
Pain	32.3 (180)	60.0 (331)	45.8 (511)	35.6	
Never visited	66.6 (371)	36.8 (203)	51.5 (574)	63.1	.000
**Dental treatment experience**					
Extraction only	18.3 (102)	32.6 (180)	25.3 (282)	20.0	
Others	5.6 (31)	11.4 (63)	8.4 (94)	6.3	.000
**Seek professional therapy for toothache**	18.0 (100)	38.6 (213)	28.1 (313)	20.4	.000

* 'Total sample' (n = 1109) includes all the school children who participated in the study while the '*representative *sample' (n = 1109) refers to the results after weighting according to the school children distribution between the two school sectors (Public: Private = 7:1)

# P value for Chi-Square test to compare proportions of sociodemographic characteristics between school sectors, public and private

The questionnaire included variables describing oral hygiene habits and attitudes such as perceived oral health and satisfaction with oral health status on 4 points Likert scales, ranging from 'very good' and 'good' (interpreted as good) to 'bad' and 'very bad' (interpreted as bad) and 'very satisfied' and 'satisfied' (interpreted as satisfied) to 'not satisfied' and 'not satisfied at all' (interpreted as not satisfied), respectively. Tooth brushing habits were reported with respect to frequency; regular (everyday once or more, once every second day, once every third day, once a week) and irregular. Also reported were the tools used for brushing (tooth brush, miswak, finger), agents used with brushing (tooth paste, water, other). Dental history was recorded based on history of visit to the dental clinic (have you visited a dental clinic before), reason for dentist visit (follow-up, pain, other), dental floss use (yes, no) and reason behind not using the dental floss (never heard of flossing, no access to dental floss, do not feel it is important, do not know how to use it, other). Dental treatment experience was dichotomised into 'extraction' (simple treatment) and others (filling, fissure sealant, orthodontic treatment, space maintainer, other). They reported toothache management as 'professional' when children sought care for toothache at a dental health care facility, hospital or private clinic and 'home management' when the child handled the toothache at home by traditional methods.

### Statistical analyses

Analyses was done using STATA version 10 to adjust for cluster sampling, marking the strata as the locality and the sampling unit as the school and the unit of analysis being the schoolchild. Multivariate analysis was performed using Poisson regression reporting prevalence ratio.

#### Principal component analysis

To construct a good subset of SES predictors, household-level information on assets and education level were combined. Other indicators were ownership to the house (65%), ownership of household durable assets such as refrigerator (77%), television receiver (90%) and car (52%); materials of the dwelling structure (cement and red brick 76%), family size (more than 3 children 49%) and house size (more than one room 91%). A principal component analysis was used to define these weights. The index used was the first principal component (eigenvalue 3.6). It summarized the largest amount of information common to the variables; 35.7% of the variability in the 10 variables loading on ownership of a refrigerator (0.89), house lighting (0.84), quality house building materials (0.81), combined parental education (0.69) and ownership of television (0.64). SES was assessed by dividing the principal component into quintiles such that each household was classified as lowest, lower, low, middle and higher SES. For the sake of providing a dichotomised variable, the latter two were combined to predict 'middle' SES and the earlier three for 'low' SES [15].

### Ethical approval

Written permission to conduct the study was obtained from the Ministry of Health and Ministry of Education in Khartoum, the local administration authorities and from the school authorities on behalf of the children. Procedures for obtaining consent and ensuring confidentiality were approved by the ethical research committee in the Sudan.

## Results

### Test-retest reliability

The questionnaire was reintroduced to a sample of 20 randomly selected 12-year-old school children. The test-retest time was 10 days. Kappa values for test-retest of the questionnaire ranged from 0.55 (knowledge) to 0.97 (wealth index). The Kappa value for DMFT was 0.83 for 45 re-examined children with a 14 day interval. These values are in the interval from moderate to substantial agreement according to Landis and Koch [16].

### Sociodemographic characteristics

The response rate was 99%. Most 12-year-old children attended 6^th ^grade in public schools (49%) (range 2–8), and 7^th ^grade in private schools (76%) (range 5–8). Four children (0.4%) reported 'no tooth-brushing', 64% reported brushing their teeth at least once a day, 25% twice a day and 5% more than twice a day. Two percent used 'miswak' (natural toothbrush made from the twigs of the *Salvadora persica *tree) [17] and 97% reported using water and toothpaste. Three percent reported using dental floss and 90% never heard of the dental floss before. Fifty-two percent never visited a dentist.

It was found that public schools had a significantly higher proportion of students with no caries experience (DMFT > 0) (76.5% vs. 69.7%). Table 1 shows the percentage distribution of the children's sociodemographic characteristics in public and private schools. The proportion of children having fathers and mothers with high education was found to be significantly higher among private school attendees compared to children attending public schools: 66.7% vs. 26.9% and 40.6% vs. 13.6% respectively. Public school attendees reported 'good perception' and 'satisfaction' with oral health more than private school attendees (78.5% vs. 73% and 75% vs. 65%, respectively).

### Clinical measures

Table 2 summarizes oral health status in terms of the number of permanent teeth, dental caries experience (DMFT > 0) and dental hygiene status (GI, PI). The mean DMFT (*representative*) among all 12-year-old school children was found to be 0.42 (SD 0.92); 0.40 (SD 0.92) and 0.57 (SD 1.19) among public and private school attendees, respectively. The *representative *decayed component (DT) of the DMFT was 0.38 (90.6%), of which 82.6% affected the first permanent molars. The *representative *missing teeth component (MT) was 0.03 (6.1% of DMFT) of which 74.0% affected first permanent molars. The *representative *filled component (FT) was 0.01. The DT, MT and FT contributed 90.2%, 7.3% and 2.5% to the DMFT in Public school attendees, compared to 86.0%, 5.3% and 8.7% respectively to the DMFT in private school attendees. Furthermore, the SiC was 1.4 for the adjusted sample and 1.6 and 1.4 for private and public school attendees respectively. The untreated dental caries prevalence was 30.5%. The highest DMFT was reported among girls attending private schools of Khartoum locality, 1.0 (CI 0.65 – 1.3), whereas the lowest was reported among the boys attending public school in Ombada locality, 0.18 (CI 0.05 – 0.32).

**Table 2:** Number of teeth and caries experience (DMFT > 0) among 12-years-old school children in Khartoum state, Sudan; mean (SD)

	**School children (public sector)** **Mean (SD) n = 557**	**School children (private sector)** **Mean (SD)** **n = 552**	**Total sample** **Mean (SD)** **n = 1109**	***Representative *sample** **Mean (SD)**
**PM**	24 (4.8)	24 (4.9)	24 (4.9)	24 (4.9)
				
**DT**	0.37 (0.83)*	0.49 (1.09)*	0.43 (0.97)	0.38 (0.87)
**MT**	0.03 (0.25)*	0.03 (0.19)*	0.03 (0.23)	0.03 (0.25)
**FT**	0.01 (0.09)*	0.05 (0.34)*	0.03 (0.25)	0.014 (0.15)
**Caries experience # in first molars only**	0.31 (0.72)*	0.37 (0.75)*	(0.34) (0.73)	0.32 (0.72)
**DMFT**	0.40 (0.92)*	0.57 (1.19)*	0.49 (1.06)	0.42 (1.0)
**SiC**	1.40	1.60	1.40	1.40
**GI**	1.05 (0.13)*	1.04 (0.13)*	1.04 (0.13)	1.05 (0.13)
**PI**	1.30 (0.36)*	1.20 (0.30)*	1.30 (0.33)	1.30 (0.35)

* P-value less than 0.05 Mann-Whitney to test difference between means of two independent groups

# DMFT > 0

PM: No. of Permanent teeth, DT: Decayed component, MT: Missing teeth component, FT: Filled teeth component, DMFT: Decayed, missing and filled teeth index, SiC: Significant caries index, GI: Gingival index, PI: Plaque index

The mean *representative *GI for the six index teeth (16, 12, 24, 36, 32, 44) was found to be 1.05 (CI 1.03 – 1.07) and the mean *representative *PI was 1.30 (CI 1.22 – 1.38). Children diagnosed with fluorosis were 11.9% of the examined sample.

There was no significant difference between genders regarding the clinically examined oral health parameters.

### Multivariate analysis

The Poisson regression model predicting caries experience from ten independent variables was statistically significant p < 0.001, chi square = 52.73, Nagelkerke R^2 ^= 0.015 and showed significant association with higher SES. Table 3 shows results from each of the unadjusted (for cluster sampling) bivariate analysis and Poisson regression analysis after adjusting for the cluster effects from the different localities. This test is a preferable alternative to logistic regression for the analysis of cross-sectional studies with binary outcomes and is able to report prevalence ratios. [18].

**Table 3:** Bivariate analysis and Poisson regression (adjusted for cluster sampling) against caries experience (DMFT > 0) with prevalence ratio (IRR) and 95% confidence interval (CI) (n = 1109)

**Sociodemographic data**	**Bivariate analysis** **IRR (CI)**	**Multivariate analysis after adjusting for cluster analysis** **IRR (CI) #**
**School sector**		
Public		1
Private	1.3 (1.00–1.64)*	1.1 (0.86–1.42)
**Socioeconomic status**		
Low		1
Middle	1.4 (1.14–1.69)*	1.23 (1.02–1.47) *
**Gender**		
Boy		1
Girl	1.0 (0.83 – 1.30)	1.1 (0.86–1.30)
**Locality**		
Urban		
Remote	1.1 (0.8–1.4)	0.90 (0.71–1.10)
**Father education**		
Low		1
High	1.4 (1.01–1.82)*	1.13 (0.80–1.6)

**Clinical parameters**		
**Mean GI index**		
Score ≤ 1		1
Score > 1	1.03 (0.81–1.30)	0.99 (0.76–1.28)
**Mean PI index**		
Score ≤ 1		1
Score > 1	1.00 (0.77–1.26)	1.04 (0.79–1.40)
**Flourosis**		
Dean's Index score		1
> 1	1.0 (0.75–1.47)	0.98 (0.66–1.45)
		
**Oral hygiene habits**		

**Tooth-brushing frequency**		
Irregular		1
Daily	1.0 (0.92–1.16)	1.04 (0.93–1.16)
**Dentist visit**		
No		1
Yes	0.6 (0.54–0.75)*	0.69 (0.58–0.82)

*p < 0.05

# Poisson regression model: p < 0.001, chi square = 52.73, Nagelkerke R^2 ^= 0.0152

## Discussion

This study reported results that can be generalized to all 12-year-old school children residing in Khartoum state. This state was selected for its unique cosmopolitan qualities. Sudanese migrate hitherto being the capital for better living opportunities and owing to internal displacement resulting from war conditions and drought. This index age enabled collation to previous studies about the Sudan and others, however, comparison of DMFT values was managed with care, acknowledging the different criteria for inclusion and caries diagnosis, dentition included (deciduous/permanent), different field conditions and criteria for handling questionable caries adopted by the following studies.

Results in this study have concluded that the prevalence of dental caries was low (DMFT 0.4). In 1966 the DMFT of 10 to 14-year-old children was reported to be 0.7 in Sudanese children generally and 1.5 in Khartoum province/state specifically [19]. In 1986, Ibrahim et al reported DMFT values of 6 to 13-year-olds, in three areas within Khartoum province, classified by the authors to urban, semi-urban and rural, of 2.9, 3.2 and 2.3 respectively [3]. Sampling procedures were not clarified. Two years later, this was followed by a reported rise of DMFT to 3.2, among a randomly selected sample of school children in Omdurman locality [4]. These authors expressed concern towards an "alarming rise" which suggested that we might find an even higher DMFT value. The present study results indicate a decline.

The DMFT value may have reduced as a result of better oral hygiene and improved dietary habits. Conversely, it may have been underestimated in the field due to the use of natural sunlight for examination [20] and that questionable cases were recorded as negative for caries. A condition that applies to all studies using the DMFT tool is that the WHO criteria for dental caries diagnosis tend to underestimate the need for treatment by overlooking *small and proximal cavities *[21]. Over and above the before acknowledged limitations of the DMFT index, the caries diagnosis methodology in the previously conducted studies was not clear enough to make an absolute comparison.

In 1993, Raadal et al, using a modified WHO criteria, reported a DMFT of 0.15 among 7 to 8-year-old school children sampled randomly from the National capital [5]. Although Raadal's target age group was younger than that in this study, they have reported a decline in DMFT, similar to our findings and in accordance with the decline in other developing countries [22,23].

DMFT prevalence among the 12-year-olds of countries neighbouring the Sudan, reported after the year 2000, were obtained from the WHO Oral Health Country/Area Profile Programme. This study has shown that presently Khartoum state stands on the lower border of DMFT values, hand in hand with Tanzania and Nigeria (Lagos) who reported a DMFT of 0.3 and 0.46 [24] among 12-year-old children in 2004 and 2003/04 respectively. Saudi Arabia, on the other hand, reported a DMFT of 5.9 in 2002 [25]. (Table 4)

**Table 4:** Mean DMFT according to CAPP database WHO.

**COUNTRY**	**YEAR OF REPORT**	**DMFT**
Egypt	1991	1.2
Ethiopia	2000	1.6
Libya/Tripoli	1984	1.6
Democratic Rep. of Congo	1987 – 1991	0.4 – 1.1
Uganda	2002	0.9
Kenya	1986	0.9 – 1.8
Botswana	1981	0.5
Malawi	1992 – 1994	0.6 – 0.8
Tanzania	1994	0.3
Saudi Arabia	2002	5.9
Yemen	1987	3.1
Jordan	1995	3.3
Iraq	1995	1.6
United Arab Emirates	1995	1.6

SiC was introduced to draw attention to those individuals with the highest caries scores since the caries distribution was observed to be generally skewed. The SiC of 1.4 for the total population is more than 2 times higher than the mean DMFT for the entire sample as reported in other studies [26]. However, it was found to be well below the upper limit of SiC value of 3 set by the WHO as a global average [27].

According to the published literature on dental caries in the Sudan, the dental caries experience (proportion of individuals with DMFT > 0) is on the decline. Emslie reported in 1966 a caries experience of 57.4% among 10–14-year-old school children examined in Khartoum [19]. Baghdady et al reported in 1979 a caries experience (DMFT > 0) among a randomly selected sample of 107 12-year-old Sudanese school children in Khartoum to be 51.4% [28]. Our study found that a representative 76% of the 12-year-olds in Khartoum were caries free. This may be attributed to the improved nutrition status and hence a better health condition today compared to that encountered more than thirty years ago [29] and also to raised awareness about oral hygiene.

The average DMFT was higher among private school attendees and children from a higher SES background. Private school attendees paid more frequent visits to the dentist and were more satisfied with their oral health. This attitude may reflect an influence from their parents who were probably more aware of oral health by virtue of their higher education. Nevertheless, they still had a higher caries experience. Public school children, with their comparatively, although small, poorer oral hygiene in terms of GI and PI, did not experience caries as often. It has been reported that the diversity in caries prevalence is partly due to the variance in dietary habits, culture and oral hygiene of different communities and is thus associated with various socio-economic and biologic risk factors [30]. The dietary habits or salivary microbiology picture of these school children, which shall be discussed in a forthcoming report, may explain some of the observed difference.

The Decay component contributed the most to the DMFT indicating an unmet need for treatment. The care index of 3.3% suggested poor coverage of oral health services in Khartoum state. Oral health services in the Sudan are available to school children through governmental services via hospitals, primary health care centres and the oral school health programme for Khartoum state and private specialised clinics for child care. The provision of all is mostly based on fee for service despite the health insurance system provided, thus becoming a burden for some of the population. The Sudan has 1.7 dentists for every 100,000 individuals as of the year 2007, mostly concentrated in major cities, depriving many from professional treatment [1]. From the results of this study, the need for treatment is emphasized and a suggestion to launch oral health prevention programs via the television broadcast since almost all (90.4%) the school children had a television at home is made.

Structured questionnaires might have had limitations whereby students may tend to report socially desirable answers such as frequent tooth brushing [31]. However, objective measures, such as clinical examination, were used to overcome this limitation. Results revealed that most of the students cared to clean their teeth on a regular basis and this tallied with the clinical values of plaque accumulation. School children who had experience with dental visits reported to have good oral health more often than their counterparts with no dental visits unlike other studies in developing countries [32,33]. This suggested that school children could have perceived good oral health as a pain-free mouth although the pilot study did not indicate this misconception.

The statistical tests used after adjusting for locality and school resulted in very minor changes in IRR values and a slight widening in the confidence intervals. Any associations found from cross-sectional observational data like this are at best suggestive of causal relationships. Nonetheless, it may be concluded that in the present study SES among school children is directly associated with caries experience. Children with higher SES reported higher DMFT values but less GI and PI scores. Children with higher SES may be able to purchase more sugary snacks. On the contrary, studies in Jordan and other countries have found that low SES is associated with a higher mean number of decayed and filled surfaces [34].

The Poisson regression model has captured only 1.5% of the variables associated with dental caries. A reason for this may be the skewed distribution of the caries experience in the sample and the low DMFT. This low explained variance suggests the need for further studies. These should include more oral health related predictors to derive a more definitive conclusion.

## Conclusion

All in all, the results indicate a low prevalence of dental caries among 12-year-olds concentrated in 24% of the school children, a need for treatment and highlight the impact of socioeconomic status as a risk indicator to the oral health status. This data may be of importance in the evaluation of the past and planning of future oral health prevention and treatment programmes targeting the high risk group.

## Competing interests

The authors declare that they have no competing interests.

## Authors' contributions

NMN designed the study and carried out the data collection, data analysis and writing of the article. TAT, MFA and RWA supervised the project and assisted in writing/editing of the article. All authors have read and approved the final manuscript.

## Pre-publication history

The pre-publication history for this paper can be accessed here:


